# Chemokine releasing particle implants for trapping circulating prostate cancer cells

**DOI:** 10.1038/s41598-020-60696-x

**Published:** 2020-03-10

**Authors:** YiHui Huang, Amirhossein Hakamivala, Shuxin Li, Ashwin Nair, Ramesh Saxena, Jer-Tsong Hsieh, Liping Tang

**Affiliations:** 1 Department of Bioengineering, the University of Texas at Arlington, Arlington, Texas 76019 USA; 2 Division of Nephrology, University of Texas Southwestern Medical Center at Dallas, 5323 Harry Hines Blvd, Dallas, TX 75390 USA; 3 Department of Urology, University of Texas Southwestern Medical Center, Dallas, TX 75390 USA; 4 Department of Biomedical Science and Environmental Biology, Kaohsiung Medical University, Kaohsiung, 807 Taiwan

**Keywords:** Cancer prevention, Chemotaxis, Cancer prevention, Thermoelectric devices and materials

## Abstract

Prostate cancer (PCa) is the most prevalent cancer in U.S. men and many other countries. Although primary PCa can be controlled with surgery or radiation, treatment options of preventing metastatic PCa are still limited. To develop a new treatment of eradicating metastatic PCa, we have created an injectable cancer trap that can actively recruit cancer cells in bloodstream. The cancer trap is composed of hyaluronic acid microparticles that have good cell and tissue compatibility and can extend the release of chemokines to 4 days *in vitro*. We find that erythropoietin (EPO) and stromal derived factor-1α can attract PCa *in vitro*. Animal results show that EPO-releasing cancer trap attracted large number of circulating PCa and significantly reduced cancer spreading to other organs compared with controls. These results support that cancer trap may serve as a unique device to sequester circulating PCa cells and subsequently reduce distant metastasis.

## Introduction

It is estimated that 174,650 new cases will be diagnosed with prostate cancer (PCa) in 2019 and almost 10% of or 31,620 patients are expected to succumb to this disease^1^. The major cause for the mortality of PCa patients is due to the onset of metastatic PCa to bone, lymph nodes, liver and lung^2–7^. There are several treatment options available. Androgen deprivation therapy has been shown to be beneficial, albeit in the stage of metastasized PCa^8^. However, cancer cells eventually acquire resistance and become incurable at the late stage^9,10^. Although radiation and targeted therapies can be very effectively to control peripheral invasion^11,12^, these approaches are not able to control those metastatic cells that might have spread into circulation^13–15^. In addition, many of current regimens therapies significantly affect the quality of life of patients by causing various side effects^16–18^. It is, therefore, of utmost importance to develop new strategies that can diminish PCa metastasis with minimal side effects.

We believe that a new strategy should be developed to attract circulating PCa cells and thus to reduce PCa spreading. Inspired by the success of roach motels that control cockroach infestations, our design is to attract migrating cancer cells using specific chemokines that can preferentially attract metastatic cancer cells into “roach motel-like” cancer traps. Our work has previously shown that implant-mediated inflammatory responses can enhance the recruitment of many different cancer cells, including PCa^19^. Our goal is to establish a new technique to attract metastatic PCa cells without eliciting inflammatory reaction. To achieve this goal, we determine that the cancer trap should possess the following three characteristics: good cell/tissue compatibility, able to be injected into subcutaneous cavity, and able to create a localized chemokine/growth factor gradience via controlled release. With these attributes, the cancer trap can recruit circulating cancer cells over time with minimal foreign body responses and surgical trauma.

These designed traps are composed of degradable, injectable polymeric microparticles capable of releasing bait (cancer specific chemokines). The injectable microparticles are made of hyaluronic acid (HA), a natural material with great biocompatibility and biodegradability^20,21^. Two chemokines/growth factors, stromal derived factor-1α (SDF-1α) and erythropoietin (EPO), are included as the “bait” based on the following early observations that find SDF-1α, and EPO to affect cancer cell migration^19,22,23^. In addition, EPO is found to promote the recruitment of a wide variety of cancer cells, including prostate, melanoma, lung, and breast^19^.

This work summarizes our effort on the design and characterization of the PCa cancer trap. HA microparticles were synthesized and used as the base of our cancer trap. We first investigated the physical properties, drug releasing kinetics and cytotoxicity of HA particles. To assess the ability of the cancer trap to preferentially attract metastatic PCa cells, we used both PC3 (poorly-metastatic) and DAB2IP-knockdown PC3 (highly-metastatic, abbreviated as KD) cells^24^. During cancer progression, circulating tumor cells detach from the primary tumor, arrest in capillaries, and extravasate and grow^25^. Due to the difficulty of counting the number of cancer cells that shed from the primary tumor and grow in secondary organs, intravenous (IV) administration of cancer cells is commonly used as an *in vivo* model to assess the extent of cancer metastasis instead of spontaneous model (orthotopic model)^26–32^. Using the intravenous cancer cell implantation model, we tested the efficacy of the cancer trap implantation to recruit and then to reduce the biodistribution of intravenously inoculated PCa cells *in vivo*. Finally, by comparing the PCa cell biodistribution in animals with or without cancer trap implants, we assessed the influence of cancer trap implant on cancer cell metastasis.

## Results

### Chemotactic activities of different chemokines and growth factors

**Figure 1:**
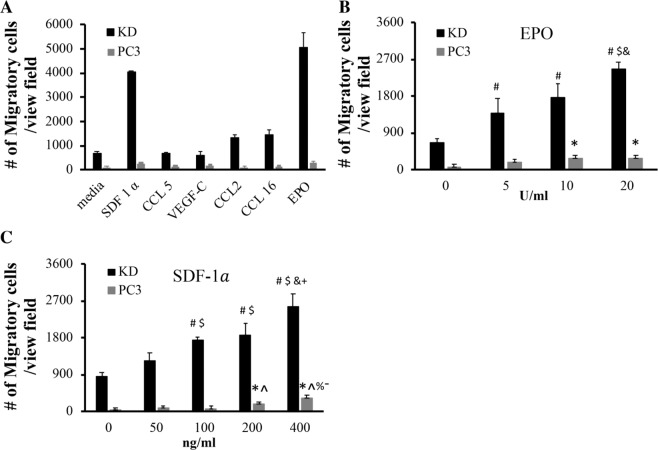
The effects of chemokines on cancer migration. The *in vitro* migration of PCa cells under different chemokines and growth factors was determined using Transwell system. The studies were carried out using either PC3 or KD cells. (**A**) Migration of KD cells treated with different chemokines and growth factors –EPO (100 U/ml), SDF-1α (100 ng/ml), CCL5 (100 ng/ml), VEGF-C (100 ng/ml), CCL2 (100 ng/ml) and CCL16 (100 ng/ml) was determined. (**B**) The influence of different concentrations of EPO (ANOVA p < 0.05 among groups of KD and PC3 cells. #, $ and & indicate p < 0.05 versus 0, 5 and 10 U/ml in KD cells and * indicates p < 0.05 versus 0 U/ml in PC3 cells, respectively.) and (**C**) SDF-1α (ANOVA p < 0.05 among groups of KD and PC3 cells. #, $, & and + indicate p < 0.05 versus 0, 50, 100 and 200 ng/ml in KD cells and *, ^, % and - indicate p < 0.05 versus 0, 50, 100 and 200 ng/ml in PC3 cells, respectively.) on the migration of KD and PC3 cells. Data are mean ± SD (*n* = 5). Experiments were confirmed statistically using ANOVA with Tukey-Kramer test.

To explore of the idea of fabricating cancer traps for PCa cells, our first task was to identify the chemokines/growth factors that are potent in promoting PCa recruitment. Based on literature search, EPO^19^, SDF-1α^23^, CCL5^33^, VEGF-C^34^, CCL2^35^ and CCL16^36,37^ were selected as potential candidates. Using Transwell cell migration system, we first determined the chemotactic ability of EPO (100 U/ml), SDF-1α (100 ng/ml), CCL5 (100 ng/ml), VEGF-C (100 ng/ml), CCL2 (100 ng/ml), and CCL16 (100 ng/ml) using highly-metastatic KD cells and poorly-metastatic PC3 cells. The concentrations for each chemokines and growth factors with highest chemotactic activities were chosen based on the manufacturer's information. Our results have shown that, as expected, KD cells are more sensitive to all biomolecules than parental PC3 cells at any given concentrations. Moreover, SDF-1α and EPO are the most potent cytokines of inducing the migration of KD cells (Fig. 1A). Subsequent studies were carried out to demonstrate the effect of EPO (Fig. 1B) and SDF-1α (Fig. 1C) on the migratory ability of KD compared with PC3 cells in a dose-dependent manner.

### Characterization of HA particles

**Figure 2:**
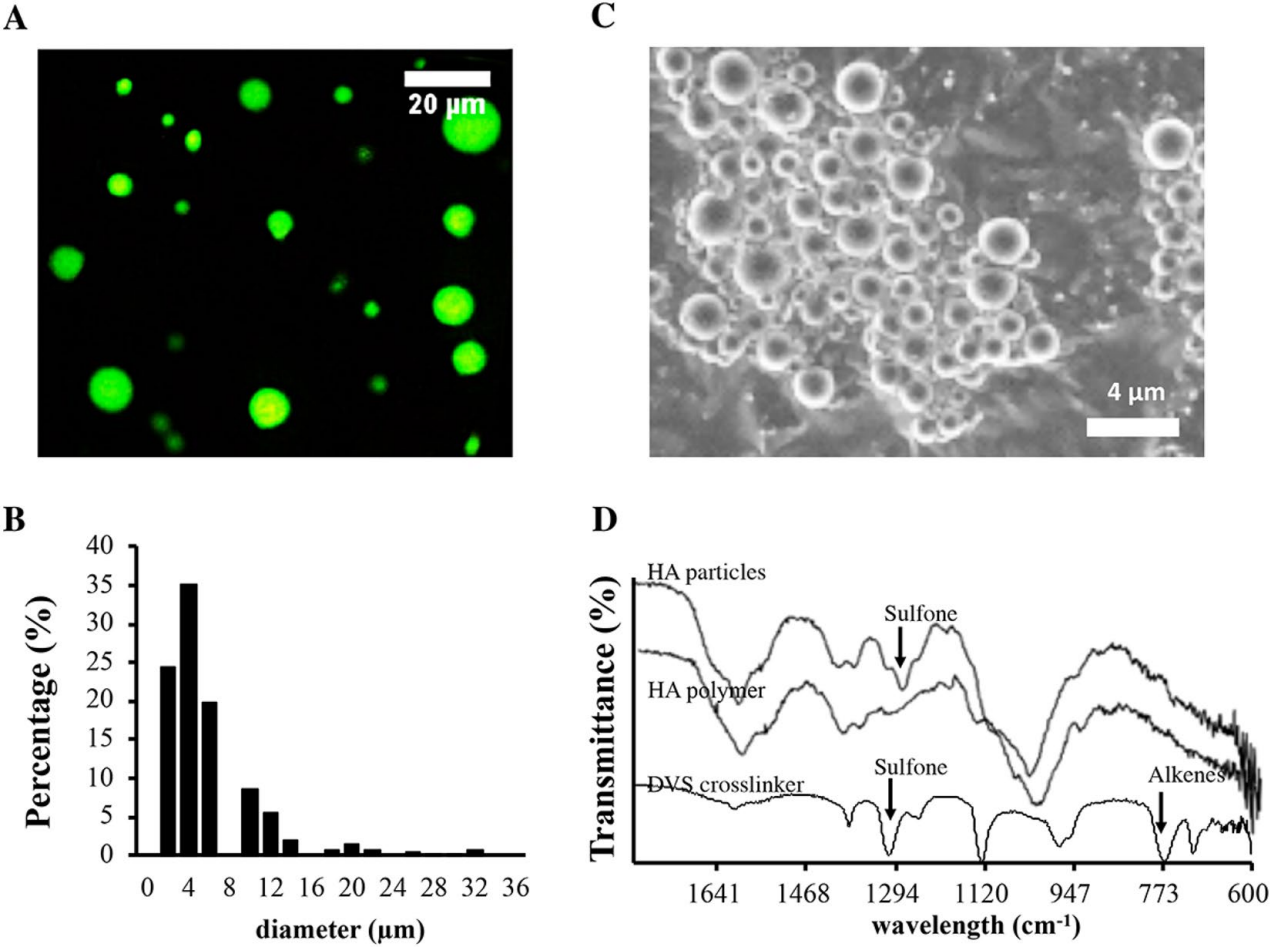
Properties of hyaluronic acid particles. The physical and chemical properties of hyaluronic acid (HA) particle with high crosslinking density (DVS: HA = 6.33:1) were characterized. (**A**) Morphology and (**B**) size distribution of HA particles were documented under fluorescence microscope. The sizes of 200 HA particles were compiled to determine the size distribution of HA particles. (**C**) Scanning electron microscope images of HA particles. (**D**) IR spectrum of HA particles, HA (700 K) polymers and DVS crosslinker. It illustrates that stretching vibration of sulfone (≈1300 cm^−1^) appears and bending vibration of alkenes (≈780 cm^−1^) disappears after HA polymers are crosslinked with DVS.

HA microparticles were fabricated and used as a chemokine reservoir of cancer trap. To reduce particle migration and cell internalization, the micron-sized scaffolds were fabricated using an emulsion polymerization technique with DVS (Divinyl Sulfone) used as a crosslinker (DVS:HA = 6.33:1 molar ratio). The microparticles have spherical appearance under fluorescence microscope and scanning electron microscopy (Fig. 2A,C). A majority of the HA particles have sizes ranging from 1.6–12 µm (Fig. 2B). From the FTIR spectrum of synthesized HA particles, we find that the stretching vibration of sulfone (≈1300 cm^−1^) appears and bending vibration of alkenes (≈780 cm^−1^) disappears on the particles after HA polymers are crosslinked by DVS (Fig. 2D).

### Slow release property, loading capacity and cell and tissue compatibility of HA particles

**Figure 3:**
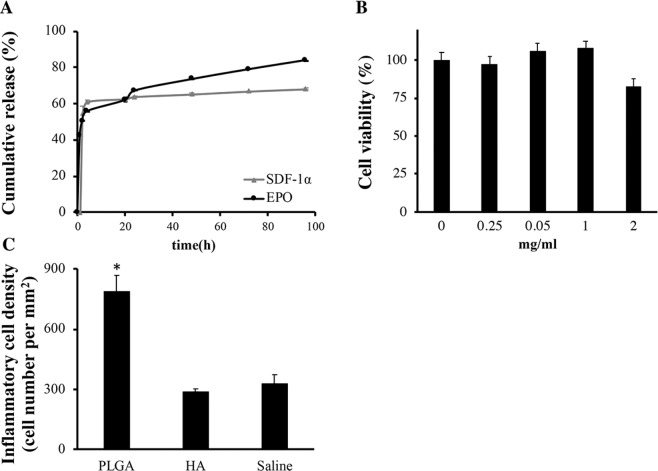
Slow release property and cell/tissue compatibility of HA particles. The slow release property, cell and tissue compatibility of hyaluronic acid (HA) particles with crosslinking densities (DVS: HA = 6.33:1, labeled as “HA”) were characterized. (**A**) The release rate of Cy5 labeled EPO or SDF-1α (Cy5-EPO or Cy5-SDF-1α) was quantified *in vitro*. (**B**) The cell compatibility of HA particles was determined using 3T3 fibroblasts *in vitro* (*n* = 5). (**C**) The tissue compatibility of HA particles was measured *in vivo* using subcutaneous implantation mice model. The density of inflammatory cells surrounding particle implants was quantified histologically to reflect the extent of tissue compatibility of different particle implants (100x magnification). (*n* = 3) Data are mean ± SD. (Student’s t-test, *indicates p < 0.05 versus Saline group).

The chemokine loading capacity and releasing capability of HA particles were evaluated *in vitro*. The loading capacities of HA particles were 17.5 μg EPO/mg particles and 1.23 μg SDF-1α/mg particles when the initial loading concentration was 20.0 μg EPO/mg and 1.60 μg SDF-1α/mg particles, respectively. In an *in vitro* system we find that the amount of released EPO and SDF-1α reached to 55% (55 μg) and 63% (5.0 μg) loading capacity of HA particles within 4 hours. After 4 hours, EPO and SDF-1α-loaded HA particles released at a relative slower speed of 0.32% (0.32 μg)/hour and 0.08% (0.0062 μg)/hour, respectively (Fig. 3A).

We found that HA particles have no apparent toxicity up to 1 mg/mL *in vitro* (Fig. 3B). The tissue compatibility of HA particles was evaluated using an mice subcutaneous implantation model and PLGA particles were used as controls. After implantation for 2 days, we found that, in comparison to PLGA particles, HA microparticles prompted significantly less inflammatory cells accumulation (Fig. 3C). Furthermore, a low amount of CD11b^+^ inflammatory cells accumulation was observed at the HA microparticles implantation and saline injection site, suggesting that HA particles have good tissue compatibility (Supplementary Fig. 1).

### ***In vivo*assessment of cancer trap**

To investigate the capability of cancer trap, EPO-loaded and SDF-1α-loaded HA particles were administered in the subcutaneous cavity. After particle implantation for 12 hours, mice were IV injected with NIR-labeled cancer cells. The distribution of cancer cells was then monitored via NIR imaging daily for up to 5 days.

**Figure 4:**
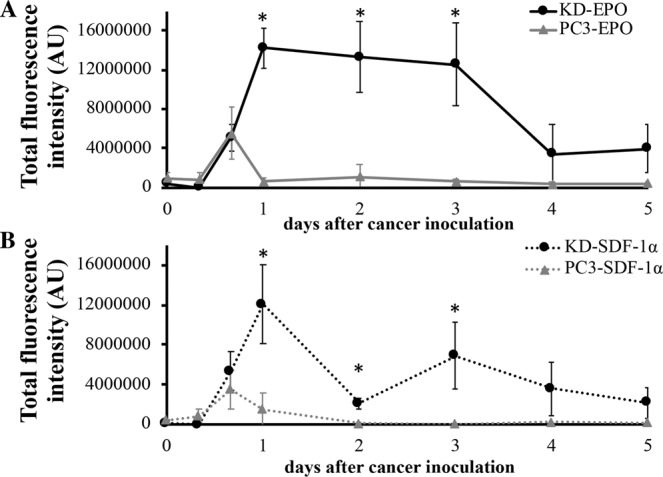
*In vivo* dynamic cancer migration pattern in cancer trap. The ability of erythropoietin (EPO) and stromal derived factor-1α (SDF-1α)-loaded HA particles to recruit PCas was evaluated for the different periods of time (8 hours to 5 days) *in vivo*. Metastatic KD and parental PC3 cells were used in this investigation. Quantitative results of cell recruitment to (**A**) EPO-loaded and (**B**) SDF-1α-loaded implants at different time points were graphed and compared. (*n* = 3) Data are mean ± SD. (Student’s t-test, *p < 0.05).

Based on NIR fluorescent intensities, we find that the chemokine-loaded HA particle implant recruited significantly more highly-metastatic KD cells than poorly-metastatic PC3 cells (Fig. 4). The maximal recruitment of KD cells to EPO-loaded HA implants was achieved in 24 hours and then lasted for 3 days. Total intensity of KD cells from the implanted site was 14,200,000 ± 2,000,000 AU/implant site (estimated 64,000 KD cells/ implant site) at Day 1 and remained at similar level until Day 3 with estimated 57,000 KD cells/ implant site. The fluorescent intensities at the implant site decreased after Day 4 (Fig. 4A). On the other hand, the maximal KD cell recruitment of SDF-1α-releasing HA was found at Day 1–12,100,000 ± 4,000,000 AU/implant site (estimated 54,000 KD cells/ implant site). The numbers of cancer cells at SDF-1α-releasing HA implant site reduced substantially (~70%) at Day 2, increased slightly at Day 3 (estimated 31,000 KD cells/implant site), and then reduced with time (Fig. 4B). The above results suggest that EPO-releasing traps have the capability to capture and retain cancer cells at the trap implant site for up to 3 days.

**Figure 5:**
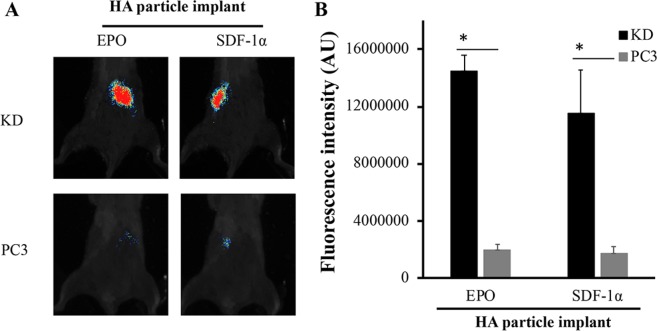
Optimization of cancer trap. The efficiency of EPO-loaded and SDF-1α-loaded particles to recruit metastatic KD and parental PC3 Qtracker-labeled cancer cells was evaluated *in vivo*. (**A**) After cancer inoculation for 36 hours, whole animal images were taken. (**B**) The fluorescent intensity at the particle implant sites was quantified. The estimated number of KD cells at EPO and SDF-1α particle implant sites are 67,000 and 53,000 cells per implant, respectively. The estimated PC3 cells at EPO and SDF-1α particle implant sites are 8,960 and 7,750 cells per implant, respectively. N = 3 in all groups. Data are shown as mean ± SD. (Student’s t-test, *p < 0.05).

To test the hypothesis, we compared the PCa cell recruitment efficiency between the PCa cell recruitment efficiency between SDF-1α- and EPO-loaded implants using the same animal model at Day 2 (36 hours after cancer inoculation). As expected, our results show that EPO implants recruited greater than 7X more KD cells than PC3 cells. In addition, SDF-1α implants attracted approximately 7X more KD cells than parental PC3 cells (Fig. 5A,B). Nevertheless, EPO implants appears to be slightly more efficient than SDF-1α implants by recruiting greater than 1.3 times more KD cells (Fig. 5A,B).

### Evaluation of the localization of PCa cells inside or surrounding of cancer trap

**Figure 6:**
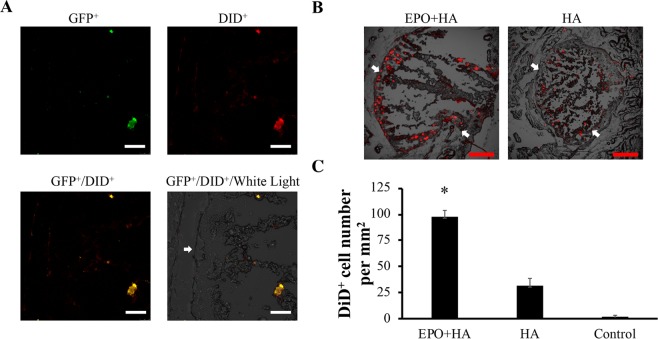
Histological analysis. Tissue sections were made and imaged to determine the distribution of KD cells in and surrounding particle implants. NIR images of EPO-loaded particles show the presence of DiD-labeled GFP expressing KD cells in and surrounding the implants. HA particles alone and particle free tissue were used as negative control. (**A**) Merged images showed DiD^+^ (red) and GFP^+^ (green) cells were co-localized in EPO implant (200x magnification). (**B**) The representative low magnification (50×) of images showed the overlapping fluorescent signals of DiD^+^ (red) KD cells in EPO-loaded implant and HA particles alone. (**C**) Quantification of DiD^+^ cells at the site of EPO-loaded particles (labeled as “EPO + HA”, 98 ± 6 cells/mm^2^), particles alone (labeled as “HA”, 31 ± 7 cells/mm^2^) and particle free tissue control (labeled as “Control”, 1 ± 2 cells/mm^2^). (*n* = 3) Data are present as mean ± SD. (Student’s t-test, *p < 0.05 compared to HA.) Scale bar: 100 µm (white) and 300 µm (red). White arrows point to the interface between the skin tissue and the implant and the areas between two arrows are implanted sites.

The recruitment of KD cells in cancer trap was examined histologically. GFP^+^ KD cells were used in this study and GFP^+^ KD cells were labeled with Vybrant DiD cell labeling dye with excitation and emission wavelengths of 649 nm and 670 nm, respectively. Tissue sections of EPO-loaded particle implants (EPO + HA) and particles alone (HA) were imaged for its NIR signals. We find significant NIR signals surrounding EPO-loaded particle implant sites (Fig. 6A). Most of the signals is localized at the interface of the particle implantation sites and surrounding host tissue. These results support the ability of EPO-loaded particles to enhance the recruitment of KD cells migration toward the implants. By overlapping NIR and fluorescent images, we determine that NIR signals coincide with GFP signals, suggesting the presence of live KD cells in and around the particle implants (Fig. 6A,B). We also find that there are significantly more NIR signals in the EPO-loaded particle implants (EPO + HA) than the particles alone (HA) (Fig. 6B). Finally, the numbers of KD cells in tissue sections were quantified. In agreement with earlier observation, we find the EPO-loaded particle implants (EPO + HA) attracted greatly than 3X more KD cells than chemokine-free particle implants (HA) and particle free tissue controls (Control) (Fig. 6C). Further studies were performed to determine whether the implantation of the EPO-loaded particles would increase EPO concentration in blood. Cy5-labeled EPO was used in the investigation. After implantation of a Cy5-EPO loaded particle implant for different periods of time (1–5 days), 10 μl heparinized blood samples were collected on daily basis. By measuring the fluorescent intensity of the blood samples, very low level of Cy5-EPO (<0.00002 unit or equivalent to 0.00001% of implanted EPO) was found in blood sample (Supplementary Fig. 2). These findings support our hypothesis that the EPO released from the particle implants may not exert any physiological effect systemically.

### Impact of cancer trap on cancer cells metastasis

**Figure 7:**
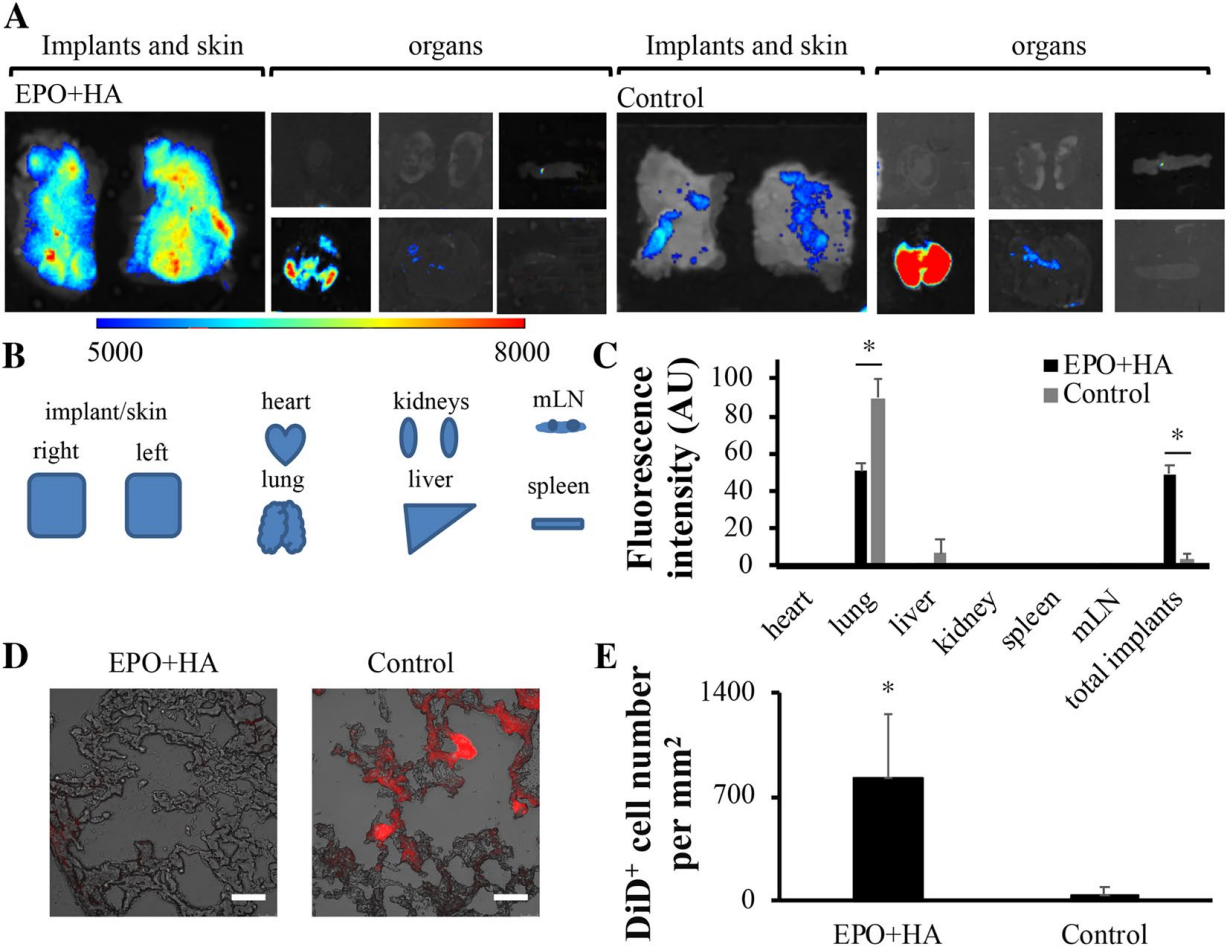
Reduction of cancer metastasis via cancer trap. Biodistribution of KD cells treated with the EPO-loaded particles or untreated control was quantified based on the measurement of organ-specific fluorescent intensities. To observe the biodistribution, NIR-labeled cancer cells were administered intravenously 12 hours following subcutaneous particles implantation injection or no treatment. (**A**) NIR imaging of internal organs were imaged 36 hours after cell administration. (**B**) A depiction of the organ arrangement. (**C**) The percentages of the cell distribution in different organs were calculated based on the individual organ fluorescent intensity divided by total internal organ fluorescent intensities. (**D**) Histological images of metastatic lung showed the accumulation of KD cells in lung (400X magnification). Scale bar: 50 µm (**E**) The lung tissue sections were used to quantify DiD^+^ cancer cells in lung sections from animals with EPO-loaded particles (EPO + HA) (829 ± 429.6) vs. untreated control (Control) (44 ± 49.8) groups. (*n* = 4) Data are mean ± SD. (Student’s t-test, *p < 0.05) Mesenteric lymph nodes is abbreviated to mLN.

To study the impact of cancer traps on cancer metastasis, EPO-loaded particle implants (EPO + HA) or particle free tissue control (Control) were implanted subcutaneously (2 implants per animal, 100 µl/site) on the back of mice. After inoculation of KD cells for 36 hours, animals were sacrificed, and all internal organs were imaged using Kodak *in vivo* imaging system (Fig. 7A,B). The fluorescent intensities of different internal organs were then quantified to reflect the progression of PCa-associated organ metastasis. Noticeably, we found that EPO particles implants significantly reduced KD cell accumulation in lung (51 ± 4%, p < 0.05) compared to control (lung: 90 ± 11%) within 36 hours (Fig. 7C). These results suggest that cancer trap device can mitigate the incidence of cancer cell metastasis via circulation. Histological study also uncovers that significant less KD cells were found in lung sections with EPO-loaded implants (EPO+HA; estimated 820±430 KD cells/mm^2^ view field) than those with untreated control (Control; estimated 44 ± 50 KD cells/mm^2^ view field) (Fig. 7D,E). This result supports that cancer traps may lure cancer cells away from circulation and indirectly reduce cancer spreading and/or metastasis. Our flow cytometry results show that there is 5.3% of KD cells are EPOR^+^ cells while only 1.4% of PC3 cells express EPOR (Supplementary Fig. 3). Such difference is not associated with EPO treatment, since the expression of EPOR among KD and PC3 cells do not change after 48 hours EPO incubation. These results support the hypothesis that the upregulation of EPOR on KD cells may not responsible for the preferential recruitment of the cells to EPO-releasing cancer trap.

## Discussion

Metastasis remains a challenging clinical problem that accounts for the majority of cancer mortality. Tumor progression towards metastasis is governed by a complex multi-step process whereby tumor cells dissociate from their primary site of growth, invade surrounding tissues, intravasate into a blood vessel or lymphatic vessel, survive in circulation, adhere to and extravasate from the vessel and form a new tumor at secondary site^24,38–40^. Our *in vitro* and *in vivo* studies have demonstrated that EPO and SDF-1α are the most capable of luring metastatic PCa. These results are supported by several recent observations. Specifically, EPO and SDF-1α have been shown to recruit PCa cells^19,41^. It should be noted that our study does not exclude the possibility that other types of cytokines and chemokines may also participate in the migration of PCa. For example, CCL2 facilitates PCa cell growth and bone metastasis^35^.

HA particles were employed to form the cancer trap based on these previous observations. Firstly, since hyaluronic acid dermal filler was approved by FDA in 2008, studies have shown that biodegradable HA has been proven to have good safety and cell/tissue compatibility^21,42^. Secondly, HA implants were found to induce minimal inflammatory responses due to their nonimmunogenic properties^43,44^. Thirdly, HA particles can be easily mixed with any chemokine in aqueous solution with denaturing its bioactivity minimally^45^. Fourth, degradation rate of HA particles is tunable via crosslinking density. Recent research showed that the maximum duration of HA-based dermal filler reaches up to 12 months and the increase of chemical crosslink density slows down degradation rate for short-term cancer treatment^44,46^. Finally, since HA particles don’t respond to the changes of the environment, diffusion, degradation or osmotic pressure are believed to be the main factors to drive the release of drug and protein from HA particles^47^.

To examine the effect of the implants, we focus on the behavior of migrating tumor cells that travel from the circulatory system to distant organs or our cancer traps. Compared to the orthotopic model or intratibial injection model, using the intravenous injection model as an experimental metastases model is more suitable to determine the efficacy of our cancer traps because of the reproductivity and the consistency of this model^48^. The intravenous injection model demonstrates the similarity of cancer cell distribution in mice and PCa patients, except in bone metastasis^6,49^. However, we create a chemokine-releasing implant made of soft natural material to prove our hypothesis that our cell-free cancer trap has the potential to compete with organ metastasis once the cancer cells enter the circulation system *in vivo*, after our previous study elucidates the mechanism of cancer migration from an *in vitro* artificial lymph node model^50^. Bone metastasis occurs in stiff bone structure with various types of cells (osteoclast, osteoblast) and chemokines (TGFβ, RANKL and VEGF)^51,52^. It is more complicated to cure bone metastasis than cancer extravasation from blood to soft organs. Since most of cancer cells migrate through circulation system or lymph nodes, in this study we emphasize the development of a universal and tunable cancer trap for different kinds of cancers.

Our findings on the preferential recruitment of circulating PCa cells to the implanted cancer traps are supported by the results shown in many previous publications^50,53–56^. Briefly, human bone marrow stromal cells seeded porous hydrogel scaffolds are found to form a vascularized niche in mice and also to recruit human circulating tumor cells released from an orthotopic prostate tumor xenograft^53^. Electrospun fiber networks have been shown to trap cancer cells from human blood^54^. Metastatic lymph nodes are shown recently to promote the migration of KD cells but not PC3^50^. Porous scaffolds made of poly(lactide-co-glycolide) have been found to recruit breast cancer cells and subsequently reduce the tumor burden within solid organs^55^ and animal survival^56^.

Our results show that the cancer trap not only recruits metastatic cancer cells but also decrease the accumulation of PCa cells in the lung and liver. These findings support that cancer trap implantation may be able to reduce PCa metastasis and, perhaps, prolong the survival of PCa patients. In fact, it is well established that patients with visceral metastases have higher mortality rate^49,57,58^. In addition, metastatic organs of PCa such as lung, liver, pleura and bone are more commonly found among patients^3,49^. By repeated administration of cancer traps, it is possible to trap a large number of circulating PCa in the subcutaneous space and, thus, indirectly delaying their spread throughout the body. In addition, using localized radiation and/or chemotherapy in conjunction with the cancer traps, a cancer therapy may be developed to eradicate metastatic PCa cells. Finally, it is well established that cancer takes years to become metastatic and long-term treatment might be needed to achieve the desired therapeutic outcome. For long-term treatment, it is possible to create a slow release hydrogel implant to extend drug release up to months^59,60^. Multiple treatment cycles with cancer traps on high-risk patients may be required yearly, along with frequent follow-up cares and monitoring. For late stage cancer patients, cancer trap can be used as a treatment option to eradicate migrating cancer cells during the localized treatments. Finally, the cancer trap treatment may not only avoid cancer recurrence but also work to actively remove circulating tumor cells during the treatment.

## Conclusion

We have developed a new cancer trap using injectable HA-microparticles that can release different chemokines/growth factor to preferentially attract circulating and metastatic PCa cells. Among chemokines tested, EPO and SDF-1α are the most potent cytokines to recruit metastatic PCa cells *in vitro*. From an animal model injected with metastatic PCa cells intravenously, subcutaneously implanted cancer traps are found to able to attract significant amount of circulating PCa cells and further reduce the presence of circulating PCa cells in several visceral organs, including lung. These results support the potential of cancer traps used in patients with metastatic PCa to reduce or prevent the incidence of distant metastasis.

## Material and Method

### Materials

HA (sodium salt, 700KDa) was purchased from Lifecore Biomedical (Chaska, MN, USA). 1-heptanol (1-HP) and Divinyl sulfone (DVS, crosslinker) were obtained from Sigma-Aldrich (St. Louis, MO, USA). Isooctane, dioctyl sulfosuccinate sodium salt (AOT), N-Hydroxysuccinimide (NHS) and 1-Ethyl-3-(3-dimethylaminopropyl) carbodiimide (EDC) were obtained from Fisher Scientific (Waltham, MA, USA). EPO (Epogen® Alpha) was purchased from Amgen Inc. (Thousand Oak, CA, USA). The rest of chemokines were purchased from Biolegend (San Diego, CA, USA).

### Preparation of hyaluronic acid microparticles

HA microparticles were synthesized via a water-in-oil microemulsion process as described earlier with minor revision^61^. Briefly, HA aqueous solution (3 mL, 1.5 wt % in 0.2 M NaOH) was added dropwise into a 50 mL oil phase solution (isooctane + 0.2 M AOT + 0.04 M 1-HP) and then DVS (15 mM) under homogenization at 28,000 rpm for 5 minutes using OMNI GLH homogenizer (OMNI international, GA, USA). The reaction was allowed to continue under a vigorous stirring (2,200 rpm for 1 hour) at the room temperature. The reaction was then stopped with the addition of 3 ml of HCl (0.2 M). The HA microparticles were collected via precipitation in acetone. The crude HA microparticles were then washed consequently with deionized (DI) water, ethanol and acetone. The purified HA microparticles were completely re-dispersed in DI water and then lyophilized for further use.

### Characterization of hyaluronic acid microparticles

To visualize the appearance of the microparticles, some of the HA microparticles were labeled with CF™488 A dye (Biotium, Inc., Fremont, CA, USA) as described earlier^62^. The structure of HA particle is composed by a dense sphere shell and a loose interior to offer a space that contains aqueous solution^63–65^. The microparticle morphology was observed and the images were captured using a Leica fluorescence microscope (Leica Microsystems, Germany) equipped with a Nikon E500 Camera (8.4 V, 0.9 A, Nikon Corporation, Japan). The obtained images were used to determine the average size and distribution of particles using Image J^66^. The degree of swelling is also tunable by varying the crosslinking densities^67,68^. Chemokine loading capacity and release kinetic were determined *in vitro* as mentioned in previous publication^69^. Briefly, Cy5-labelling chemokine (EPO: 100 μg, SDF-1α: 8 μg) was loaded into freeze-dried HA microparticles (5 mg) by a “breathing-in” method^70^ when most of water solution accumulates in the interior space of HA particles and at the same time the size of particles is swelling to bring more solution into HA particles^64,67^. Subsequently, 500 μL of PBS buffer was added on the top of the HA microparticles incubated at 37 °C. At a predetermined time, the supernatant was collected, and the release medium was replaced with an equal amount of the fresh one. The amount of the released chemokine, based on a calibration curve, was measured using a microplate reader (Infinite® M200; Tecan Group Ltd, Switzerland). The cumulative release was calculated as the total amount of released chemokine at a specific time relative to initial loading amount. The *in vitro* cytotoxicity of the HA microparticles was determined by using MTT assay of 3T3 Swiss albino fibroblast cells (ATCC, Manassas, VA, USA) as described previously^71,72^. The *in vivo* toxicity of the HA microparticles was evaluated using a mouse subcutaneous implantation model as described earlier^73^.

### Cell culture and migration assay

Early study has shown that DAB2IP gene knockdown in PC3 cell, a poorly-metastatic line, increases its metastatic potential (also called as DAB2IP-knockdown PC3 cells or KD cells)^24^. These stable cell lines expressing dual reporter genes (GFP and luciferase) were maintained in RPMI1640 medium (Invitrogen, Carlsbad, CA, USA) containing 5% PBS as previous described^24^. Migration assays were performed in Transwell dishes (Corning Costar, Cambridge, MA, USA) as described earlier^73^. For tracking the cell migration *in vivo*, both PC3 and KD were labeled with Qtracker® labeling kit (Life technologies, Carlsbad, CA, USA) by following manufacture's instruction as illustrated earlier^74^. For histological study, Vybrant DiD cell labeling dye (Thermo Fisher Scientific, Waltham, MA, USA) was used to track cancer cells according to the product information for observation under microscope^75^.

### ***In vivo*cancer cell recruitment**

The animal study was designed to assess the capability of the particle implants to reduce cancer metastasis using an intravenous injection model. The intravenous injection model has been widely used as an experimental model to determine the metastasis efficacy of cancers, including PCa^26,31,32,48^. To assess the ability of chemokine-releasing HA microparticles for recruiting PC3 and KD cells, immunocompetent Balb/c mice (Taconic Biosciences, Rensselaer, NY, USA) was used in this study. The animal experiments were approved by the Animal Care and Use Committee (IACUC) at the University of Texas at Arlington in accordance with the Animal Welfare Act and Guide for the Care and Use of Laboratory Animals. Furthermore, animal procedures also comply with the Public Health Service “Policy on Humane Care and Use of Laboratory Animals”. The animal procedure is summarized below. First, HA microparticles were mixed with different kinds of chemokines prior to *in vivo* experiments. Various groups of HA microparticles (9% w/v, 100 µl/implant site) were implanted on the back of animals via a 21-gauge needle. After particle implantation for 12 hours, PCa cells (5×10^6^ cells/animal) were injected intravenously (IV) into mice. *In vivo* cell migration was monitored using Kodak *In-Vivo* Imaging System FX Pro (Carestream Health Inc., New Haven, CT, USA) as described previously^19,76^. To determine *in vivo* cell distribution, at the end of the study, internal organs were isolated and their associated fluorescence intensities were measured based on the NIR images. Finally, the extent of cancer cell recruitment and “cancer trap” biocompatibility was evaluated histologically as previous described^19^.

### Statistics

All the data were evaluated using two-tailed student t-test and presented as mean ± standard deviation. The differences among each group were compared based on ANOVA and Tukey-Kramer test. Differences were designated as statistically significant when *P* ≤ 0.05 (Student's *t*-test).

## Supplementary information

Cancer trap-Supplementary data-011020.

## Data Availability

The datasets from this study are available from the corresponding author on reasonable request.
