# Supplementary material for: Chemokine releasing particle implants for trapping circulating prostate cancer cells
Source: Sci Rep. 2020 Mar 10;10:4433. doi: 10.1038/s41598-020-60696-x (PMC7064596; doi:10.1038/s41598-020-60696-x)
Supplement: Supplementary file 1 — Cancer trap-Supplementary data-011020. [file 41598_2020_60696_MOESM1_ESM.pdf]

Chemokine releasing particle implants for trapping circulating prostate cancer cells

YiHui Huang,[1] Amirhossein Hakamivala,[1] Shuxin Li,[1] Ashwin Nair,[1] Ramesh Saxena,[2] Jer-Tsong Hsieh,[3,4] Liping Tang[1,4]*

[1]Department of Bioengineering, the University of Texas at Arlington, Arlington, Texas 76019

[2]Division of Nephrology, University of Texas Southwestern Medical Center at Dallas, 5323 Harry Hines Blvd, Dallas, TX 75390

[3]Department of Urology, University of Texas Southwestern Medical Center, Dallas, TX 75390.

[4]Department of Biomedical Science and Environmental Biology, Kaohsiung Medical University, Kaohsiung 807, Taiwan

* Corresponding author: Liping Tang, ltang@uta.edu, Phone: 817-272-7523

Supplementary Figures and Legends

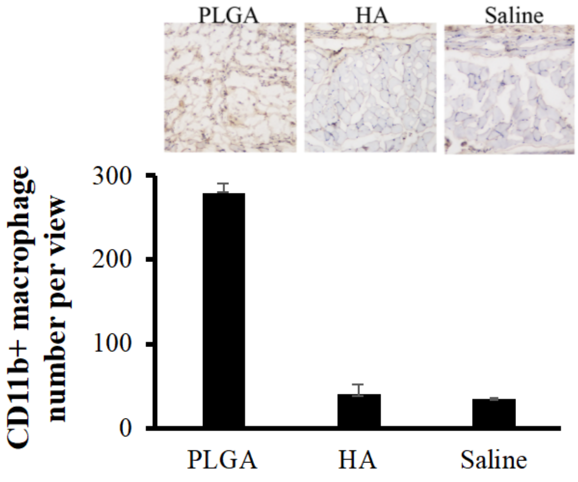

Supplementary Figure 1. Foreign body response of cancer trap. After 2-day implantation, PLGA, HA and saline samples were collected and sectioned. Immunohistochemistry was performed using CD11b antibody. CD11b$^+$ cells were counted per view after 100x magnification of IHC images were taken. (n = 3) Data are mean ± SD.

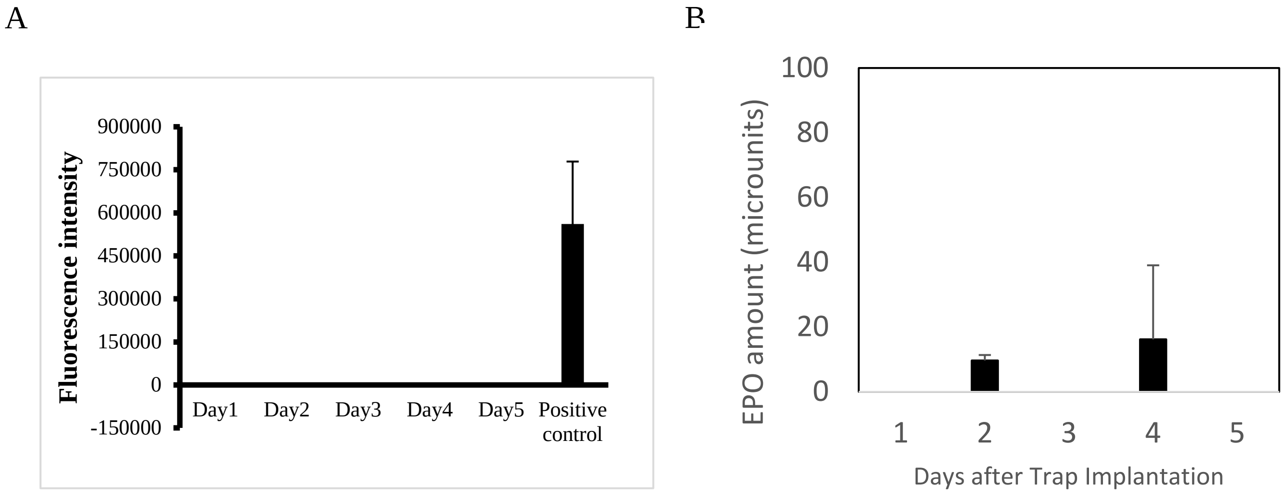

Supplementary Figure 2. Serum human EPO level. Cancer traps with Cy5-human EPO were embedded subcutaneously. Blood samples were collected continuously for 5 days. Cy5 intensity of these blood samples was measured using a microplate reader in arbitrary units (A) or in microunits. (B)

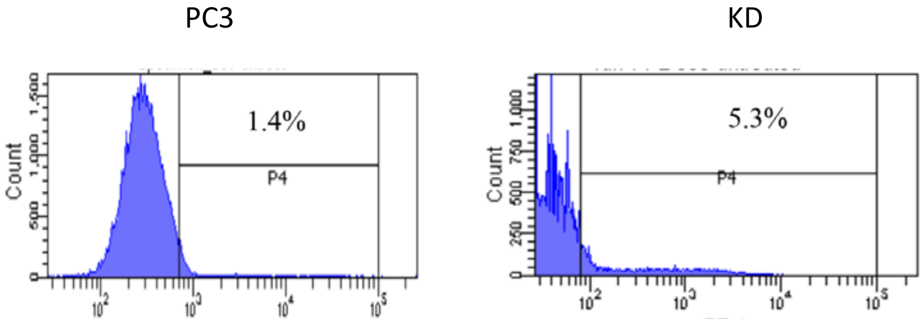

Supplementary Figure 3. Flow cytometric analysis of EPOR⁺ cells. The EPOR expression of PC3 and KD cells were detected with EPOR antibody and FITC-secondary antibody. The percentage of $EPOR^+$ cells were then calibrated using BD LSRII flow cytometer.
